# Inventory of ethical problems in mobile pre-hospital care

**DOI:** 10.1590/0034-7167-2023-0539

**Published:** 2025-01-10

**Authors:** Simone da Silva Oliveira, Mariana Oliveira Antunes Ferraz, Darci de Oliveira Santa Rosa, Kátia Santana Freitas, Elaine de Oliveira Souza, Deisy Vital dos Santos, Marisa Aparecida Amaro Malvestio

**Affiliations:** I Universidade Federal da Bahia. Salvador, Bahia, Brazil; II Universidade Estadual do Sudoeste da Bahia. Jequié, Bahia, Brazil; III Universidade Estadual de Feira de Santana. Feira de Santana, Bahia, Brazil; IV Universidade Federal do Recôncavo da Bahia. Santo Antônio de Jesus, Bahia, Brazil; V Universidade de São Paulo. São Paulo, São Paulo, Brazil

**Keywords:** Validation Studies, Bioethics, Ethic, Nurses, Emergency Relief, Estudios de Validación, Bioética, Ética, Enfermeras y Enfermeros, Socorro de Urgencia

## Abstract

**Objective:**

to construct and validate the content of an inventory of ethical problems experienced by nurses in mobile pre-hospital care.

**Method:**

a psychometric approach study, developed with the following stages: (1) instrument construction through a theoretical matrix based on deliberative bioethics, scoping review and online qualitative research; (2) content validity by judges; (3) pre-testing with Mobile Emergency Care Service nurses in various Brazilian states. For content validity analysis, the Content Validity Ratio was calculated (CVR>0.45 for judges and CVR>0.35 for the target population).

**Results:**

the instrument had 44 items, distributed across four dimensions.

**Final considerations:**

the constructed instrument presented sources of evidence of content validity, providing good psychometric measurements and constituting a useful tool for nurses’ practice in the pre-hospital setting.

## RESUMEN

**Objetivo:**

construir y validar el contenido de un inventario de problemas éticos vividos por enfermeros en la atención prehospitalaria móvil.

**Método:**

estudio con enfoque psicométrico, desarrollado con los siguientes pasos: (1) construcción del instrumento a través de una matriz teórica basada en bioética deliberativa, scoping review e investigación cualitativa en línea; (2) validez de contenido por parte de los jueces; (3) preprueba con enfermeros del Servicio Móvil de Atención de Urgencias de varios estados brasileños. Para el análisis de validez de contenido se calculó el Ratio de Validez de Contenido (CVR>0,45 para los jueces y CVR>0,35 para la población objetivo).

**Resultados:**

el instrumento contó con 44 ítems, distribuidos en cuatro dimensiones.

**Consideraciones finales:**

el instrumento construido presentó fuentes de evidencia de validez de contenido, proporcionando buenas mediciones psicométricas y constituyendo una herramienta útil para la práctica del enfermero en el ámbito prehospitalario.

## INTRODUCTION

Mobile pre-hospital care (MPHC) is defined as assistance that occurs outside intra-hospital settings by professionals who make up teams from mobile units in a work process from a multidisciplinary perspective, aiming to ensure emergency care to people, according to the degree of complexity of each case. In this service, professionals experience challenges in caring for users in the most unusual circumstances and, at times, face difficult priorities between conflicting values and norms^(1)^.

Given the context that involves unpredictability in care situations, the MPHC scenario is inviting for unusual experiences, whose complexity advocates the need for discussion about ethical problems (EPs) that emerge in nurses’ daily work.

In the context of clinical practice, ethical conflicts, which generate indecision among the professionals involved in the case, can be perceived as problems or dilemmas. The difference between EPs and dilemmas is that in the first there are several possibilities for resolving the case, while in the second decisions are always reduced to two extreme solutions^(2)^. Aware of the moral plurality that involves professional action, ethical issues classified as problems translate the phenomenon with its multiple ethical meanings and the need for dialogue and reflection in the search for responsible solutions in nursing practice^(3)^.

Thus, ethical problems that involve patients’ interest, professionals’ ideas, the organizational/management structure, decisions during user resuscitation and the interference of other people and/or professionals at the time of care, are described as frequent ethical problems in MPHC^(4)^.

In Brazil, there are instruments that have been used and adapted to measure EPs in Primary Care. The Inventory of Ethical Problems in Primary Health Care (IPE-APS - *Inventário de Problemas Éticos na Atenção Primária à Saúde*) was pioneeringly structured into three dimensions that report the phenomenon experienced by medical professionals and nurses^(5)^. It was then adapted to measure the construct in the area of child health and presented valid and reliable psychometric properties^(6)^.

It is noteworthy that validated instruments must not be presented as a mere description of ethical problems, but must direct actions to transform practice through evidence^(7)^. However, despite the relevance of the phenomenon, no studies were identified in the literature that address measurement parameters to identify EPs in the context of MPHC, highlighting a knowledge gap.

Therefore, it is considered important to analyze cultural, moral and ethical aspects intrinsic to the phenomenon, taking into account levels of healthcare. To this end, by developing an EP measurement instrument for MPHC, a new structure for ethical equations and decision-making processes in nursing is made possible.

Therefore, developing an appropriate instrument for measuring the ethical problem in a mobile pre-hospital care scenario can favor the making of sensitive and prudent ethical decisions as well as developing ethical-moral skills of nurses who work in this healthcare service^(8)^, enhancing the quality of care in emergency situations.

## OBJECTIVE

To construct and validate the content of an inventory of ethical problems experienced by nurses in mobile pre-hospital care.

## METHODS

### Ethical aspects

This study was approved by the Research Ethics Committee of the *Universidade Federal da Bahia* School of Nursing, and meets the requirements set out in Resolution 466/12 of the Brazilian National Health Council, which regulates research involving human beings, and a technical note that ensures guidelines for development research in a virtual environment. All participants filled out the Informed Consent Form (ICF).

### Study design, site and period

This is a study with a psychometric approach based on the American Educational Research Association (AERA), American Psychological Association (APA), and National Council on Measurement in Education (NCME)^(9)^ recommendations. It was developed from October 2021 to April 2022 in three stages: (1) instrument construction through the theoretical matrix, scoping review and online qualitative research; (2) content validity by a panel of judges; and (3) pre-testing with the target population.

The study is part of a doctoral thesis entitled “*Construção e validação de um inventário de problemas éticos vivenciados por enfermeiras no atendimento pré-hospitalar móvel*” ^(10)^, presented to the Graduate Program in Nursing and Health at the *Universidade Federal da Bahia*. Given the context of the COVID-19 pandemic, the research remained in an online format in the stages that involved participants, using tools such as Google Forms^®^, WhatsApp^®^ and email. It is also ensured that this modality has been proposed as a driver for reaching populations, mainly for conducting expert panels, when they are geographically distributed in different regions^(11)^.

### Population and sample: inclusion and exclusion criteria

In the first stage, online qualitative research was carried out using an intentional, non-probabilistic sampling, representing nurses from five regions of Brazil. To access this population, a national WhatsApp^®^ group of instructors of Pre-hospital Trauma Life Support (PHTLS) and Advanced Trauma Care for Nurses (ATCN) was used, of which one of the authors is a member. Participants must be a nurse in the Mobile Emergency Care Service (SAMU - *Serviço de Atendimento Móvel de Urgência*), with a minimum of one year of experience in the area.

For the second stage, we composed a committee of judges, meeting the criteria as follows: being a nurse with experience in pre-hospital and emergency, with a focus on management, research, teaching, direct care, or who were close to the EP construct and/or had expertise in validating instruments in healthcare practice. The selection of this population occurred through a simple search on the *Lattes* Platform, using the keywords “Bioethics”, “Mobile Pre-Hospital Care” and “Brazilian Nationality”. The national WhatsApp^®^ group of PHTLS and ATCN instructors was also used. To achieve greater reach in the number of participants in the event of a lack of consensus when judging the instrument, a heterogeneous composition was considered for this stage^(12)^. A number above ten judges, as associated with acceptable reliability^(13)^, met the complexity for the development of items and dimensions.

Finally, in stage 3, the target population of nurses, members of the SAMU intervention teams in the country, was selected for convenience.

### Theoretical-methodological path

The first stage of study consisted of construct theoretical and conceptual foundation, supported by Diego Gracia’s moral deliberation theoretical-methodological framework, for theoretical matrix construction, considering ethical conflicts as problems and substantiating that reality presents a complex moral plurality with a proposal of prudent alternatives capable of valuing what is involved in a clinical situation^(14)^.

For the dimension and item construction stage, a literature review was carried out in the scoping review format with the aim of mapping scientific evidence on the ethical problems experienced by nurses in MPHC. The model proposed by JBI was used to conduct all phases of this stage. To this end, the review was registered in the Open Science Framework (OSF) with DOI registration: 10.17605/OSF.IO/PS9RA^(15)^. However, the metadata was accessed from October to November 2021 in the MEDLINE/PubMed, *Literatura Latino-Americana e do Caribe em Ciências da Saúde* (LILACS), SAGE Journals, Cumulative Index to Nursing and Allied Health (CINAHL) and Web of Science databases. We considered the Descriptors of Health Sciences (DeSC), Medical Subject Headings (MeSH) and keywords “nursing”, “nurse”, “prehospital”, “ethics”, “emergency relief”, “bioethics”, “ethical dilemmas”, “prehospital”, “ambulances”, combined with each other with the Boolean operators “AND” and “OR”. Scientific texts made available in full were included, without design or language restrictions^(15)^.

In stage 1, in qualitative research, carried out in November and December 2021, an online questionnaire was applied considering the control of *etic* (researcher’s observations about the construct) and *emic* (local people’s observations about the construct) for formulating items in validity studies through the participation of the population that experiences the phenomenon^(16)^. Data collection was carried out using a questionnaire, Google Forms^®^, composed of instructions for completing the questionnaire, data relating to personal and professional characterization and dimension presentation, in addition to items highlighted in the literature review, for subsequent identification of their occurrence by respondents through multiple choice. We also chose a text space for participants to describe other EPs experienced in MPHC practice. The report at this stage was guided by the COnsolidated criteria for REporting Qualitative research (COREQ)^(17)^.

Armed with this information, a research group composed of three researchers with expertise in ethics, bioethics and development of health instruments analyzed each item and dimension to structure the instrument.

For stage 2, the content validity process was included. We sought to assess the scope of the dimensions as well as item clarity, relevance and overall assessment, obtaining opinions from judges on the instrument’s semantic and operational adequacy. An electronic form via Google Forms^®^ with general instructions for participants, a form on respondent personal, professional and academic characteristics were used, as well as initial questions related to dimension assessment. The criterion for coverage was considered when assessing whether all items covered the pre-defined dimensions and represented the latent variable.

As for the items, assessment occurred individually through judges’ judgment regarding relevance (observing whether the items reflected behavior that expressed the ethical problem that was intended to be measured) and clarity (checking whether the wording of an item was understandable) criteria. The responses followed a dichotomous scale; for this stage of study, a score of zero was used for “no” answers and a score of one for “yes” answers. It was also possible to carry out a general assessment of the instrument based on the following elements: presentation of items and dimensions; guidelines for the target population; and the response scale proposed for the instrument. The requirement of the operational scenario in its development phase being essential to instrument assessment was met^(18)^.

After adjusting the instrument, stage 3 was carried out, through pre-testing with the target population, which took place in April 2022. A preliminary version, with validated content, was tested by nurses who assessed item clarity and understanding, in addition to the operational aspects for instrument applicability in the target population. The report of the quantitative stages was conducted based on STrengthening the Reporting of OBservational studies in Epidemiology (STROBE) items^(19)^.

### Analysis of results, and statistics

To present the scoping review data, PRISMA-Scr was used, which offers clarity and transparency in data presentation from the scoping review carried out^(20)^.

For organization and analysis, data from the quantitative study stages were tabulated in Microsoft Excel^®^, and then assessed. In the content validity analysis process, the Content Validity Ratio (CVR) was calculated, created by Lawshe and calculated using the formula CVR = (n_e_ - N/2) / (N/2), as n_e_ is the number of evaluators who judged the item positively (clear/relevant) and N is the number of judges participating in the study^(21)^. The index has been indicated in the literature as it does not suffer from data inflation with the adequacy of the number of experts that make up the panel^(22)^.

Therefore, CVR 0.45 was considered as a cut-off point, with items with lower values being evaluated and modified or excluded according to judges’ recommendations.

Statistical Package for the Social Sciences (SPSS) version 22.0 was used to analyze continuous variables regarding measures of central tendency (mean) and dispersion (standard deviation), and participant characterization was presented by relative frequency.

## RESULTS

**Figure 1:**
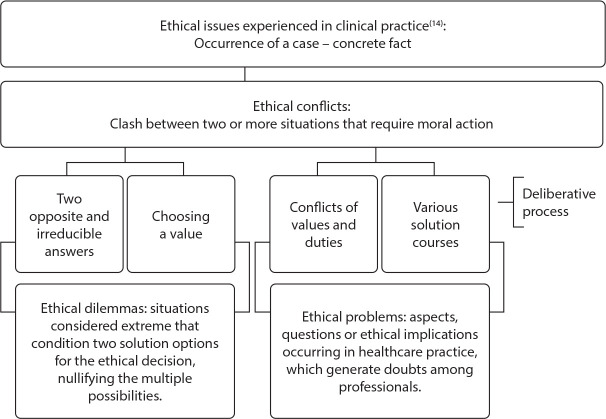
Conceptual framework of ethical problems in nurses’ clinical practice

The results of the first stage of study correspond to EP construct theoretical matrix construction, obtained through a literature review and theoretical foundation of deliberative bioethics^(14)^. Thus, a conceptual framework was proposed (Figure 1), which was revisited by the researchers during all stages of development, refinement and adjustments of dimensions and items.

The categories were read, obtained with the review and theoretical contribution, considering the conflicts of values and duties based on moral deliberation conceptual framework, to describe the ethical problems found in the MPHC scenario, and consequent construction of dimensions and items.

The scoping review reached nine texts, providing the basis for the first stage of instrument development. Thus, four dimensions emerged, namely: Dimension 1: Ethical problems arising from relationships with users and/or family members at the time of care provided by MPHC teams; Dimension 2: Intra and inter-team ethical problems of RUE components; Dimension 3: Ethical problems related to the organizational structure of RUE components; Dimension 4: Ethical problems related to external factors involved in the MPHC scenario^(15)^.

Accordingly, 12 items were expanded to the operational framework, described by the target population during online survey questionnaire self-administration. Therefore, a structure with 55 items was defined for the constructed instrument called Inventory of Ethical Problems experienced by nurses in Mobile Pre-Hospital Care (IPE-APH - *Inventário de Problemas Éticos vivenciados por enfermeiras no Atendimento Pré-Hospitalar Móvel*).

Thus, the first version of the inventory went through the content validity stage by 22 experts, the majority of whom were female (72.7%), with academic degrees ranging from master’s degree (31.8%), doctoral degree (31.8 %) to specialization (31.8%). They presented a mean age of 43.7 years (SD = 8.4), training time of 18.8 years (SD = 8.2) and time working in MPHC of 17.4 years (SD = 7.6). The identified places of professional activity were ten Brazilian states, such as Acre, Bahia, Goiás, Pará, Paraná, Pernambuco, Rio Grande do Sul, Santa Catarina, São Paulo, Sergipe, and the Federal District. Of these, they have experience in care (72.7%), teaching (72.7%), management (31.8%) and research (54.5%) in pre-hospital care, ethics, bioethics or measure instrument.

During the analysis of the data obtained from assessment, the four dimensions had a critical CVR value of 0.73 for coverage, characterizing the coverage of the set of ethical problems by the latent variable. The CVR was also calculated for the items, as shown in Table 1.

**Table 1:** Content Validity Ratio for selecting items from the Inventory of Ethical Problems experienced by nurses in mobile pre-hospital care regarding relevance and clarity. Brazil, 2022 (n=22)

Dimension 1: Ethical problems arising from relationships with users and/or family members at the time of care provided by MPHC teams			
**Items**		CVR^*^	
		**R**	**C**
1	User refusal to be assisted by professionals of the opposite sex to theirs.	0.55	0.91
2	User refusal to be transported by the MPHC team.	0.55	0.82
3	Participation in procedures without user consent.	0.91	0.27
4	Disrespect for users’ right to choose.	0.91	0.73
5	Disrespect for the family’s right to choose.	0.91	0.64
6	Omission of information about the occurrence by family members.	0.73	0.64
7	Difficulty in protecting confidential information when assisting users.	0.91	0.73
8	Difficulty maintaining user privacy during service.	0.91	0.73
9	Neglect in maintaining life support in the case of a user with a poor prognosis.	1.0	0.64
10	Lack of information for users that justifies professional service actions.	0.82	0.55
11	Lack of active listening by nurses to user complaints during care.	0.73	0.73
12	Doctor’s lack of active listening to user complaints during care.	0.91	0.36
13	Judgment by MPHC nurses on religious aspects that embarrass users.	1.0	0.55
14	Judgment by MPHC nurses on sexual aspects that embarrass users.	0.91	0.55
15	Judgment by MPHC nurses on gender aspects that embarrass users.	0.82	0.73
16	Judgment by MPHC nurses on socioeconomic aspects that embarrass users.	0.82	0.45
17	Racial ethnic prejudice by MPHC nurses.	1.0	0.55
18	Prejudice towards victims of attempted suicide by MPHC nurses.	1.0	0.91
19	Disrespect between MPHC teams and reference units.	1.0	0.91
**Dimension 2:** Intra and inter-team ethical problems of RUE components			
**Items**		CVR^*^	
		**R**	**C**
20	Disrespect between nurses and other MPHC components	1.0	0.91
21	Difficulty in communicating between nurses and other professionals on the MPHC team.	0.73	0.73
22	Difficulty in communication between members of MPHC teams and reference units.	0.91	0.82
23	Non-compliance with protocol by MPHC teams.	0.91	0.64
24	Disagreement between regulatory physicians and MPHC intervention team members.	0.64	0.64
25	Lack of empathy by professionals who make up the emergency regulation center.	0.36	0.55
26	Questioning members of the advanced support team regarding medical conduct arising from regulation.	0.55	0.55
27	Technical lack of preparedness of professionals who make up MPHC teams.	0.64	0.64
28	Lack of preparedness of medical professionals who make up in-hospital during transfer of care.	0.45	0.55
29	Difficulty for nurses to prioritize care for multiple victims in disaster situations.	0.55	0.27
30	Disagreement with medical conduct when it compromises user safety.	0.82	0.55
31	Omission of conduct by the medical team during care.	0.73	0.64
32	Lack of commitment from professionals to the MPHC service.	0.73	0.55
33	Difficulty in collaboration between professionals who make up the intervention team.	0.73	0.73
34	Disagreement in therapeutic management between regulatory physicians and MPHC intervention team members.	0.64	0.64
35	Omission in recording procedures performed with users by MPHC professionals.	0.91	0.73
**Dimension 3:** Ethical problems related to the organizational structure of RUE components			
**Items**		CVR^*^	
		**R**	**C**
36	Removal of patients without indication of emergency care.	0.55	0.64
37	Delay in the advanced support team’s dispatch to service due to the risk stratification process.	0.73	0.27
38	Unavailability of beds in reference units.	0.55	0.82
39	Refusal to admit users by in-hospital teams.	0.91	0.91
40	Difficulty in the telecommunications system between the emergency regulation center and the intervention team.	0.45	0.82
41	Sending mobile units to non-serious incidents.	0.55	0.73
42	Lack of equipment for user support.	0.55	0.73
43	Shortage of inputs for user services.	0.73	0.73
44	Embezzlement of Advanced Support Unit teams.	0.73	0.82
45	Retention of mobile unit equipment with users admitted to reference units.	0.73	0.82
46	Delay in user service response time due to fleet embezzlement.	0.91	0.82
**Dimension 4:** Ethical problems related to external factors involved in the MPHC scenario			
**Items**		CVR^*^	
		**R**	**C**
47	Disrespect for preserving the team’s image through photos taken during care.	0.91	0.91
48	Disrespect for preserving the team’s image through videos obtained during care.	0.64	0.64
49	Delay in user service due to interference from members of the public at the scene of the incident.	0.73	0.73
50	Interference in user care due to the actions of other health professionals present at the scene.	0.73	0.82
51	Interference in user care due to family members present at the scene.	0.73	0.73
52	Difficulty in providing assistance to users due to delays in police support at the scene.	0.55	0.82
53	Difficulty in providing customer service due to delay in fire department support at the scene.	0.36	0.73
54	Delay in customer service due to the time taken to file a police report.	0.36	0.64
55	Exposure to situations that threaten the life of the team at the scene of the incident.	0.55	0.73

* *Content Validity Ratio; R = relevance; C = clarity.*

Due to the number of items that obtained a CVR <0.45 in relevance and clarity criteria assessment, a hierarchical assessment was chosen, prioritizing the exclusion of non-relevant items, and clarity was defined together with an in-depth analysis of the theory that supports the instrument to maintain, change or exclude the item, according to a psychometric study validated in Brazil^(23)^.

In relation to items, it was observed that the majority reached a value greater than 0.45, which meets the value recommended in the literature, according to the number of judges^(21)^. Seven items did not obtain the estimated value, among these, four were eliminated (25, 29, 53 and 54) and three were assessed (3, 13, 37).

During an extensive instrument assessment, items 13 and 37 were considered by the research team as relevant to the instrument and were therefore maintained. Item 1 had the sentence “professionals of the opposite sex to theirs” replaced by “MPHC professionals”. Item 18 was adjusted with the inclusion of “or in psychological distress”, suggested by judges. Item 3, even with a CVR of 0.91 for relevance and 0.27 for clarity, underwent an adjustment of terms with the replacement of the word “participation” by “accomplishment”.

Item 47 was modified with the addition of the words “videos or photos”, following judges’ suggestion and the reading of the research team with the understanding that, regardless of the medium used, disrespect for the preservation of the team’s image, such as EP, is obtained. In this regard, item 48 was considered and excluded from the instrument.

Items 21, 22, 26, 30 and 31, despite satisfactory indices, were considered already covered and were removed from the version of the instrument. Item 28 was adjusted by replacing the terms “medical professionals’ unpreparedness” with “professionals’ technical unpreparedness”. Item 35 had the word “omission” replaced by the term “insufficient registration”.

During the analysis, the item “Pre-judgment by professionals when assisting users of alcohol and other drugs” was added, taking into account judges’ guidelines regarding the frequent occurrence of this EP in the daily life of MPHC.

Even with item adjustments and refinement, the instrument maintained a total CVR of 0.81 for relevance and 0.71 for clarity.

Thus, after the changes suggested by the judges, the IPE-APH was structured with 45 items and submitted for assessment by the target population. In this stage, 32 nurses (65.6%) participated, most of them with specialist academic qualifications (68.8%). They presented an average age of 40.6 years (SD = 6.6), training time of 14.2 years (SD = 7.1) and time working at the MPHC of 9.6 years (SD = 4.7). Participants declared themselves mostly black (31.3% brown and 21.9% black), and the Advanced Support Unit was the type of staffing unit with the highest number of professionals (81.3%).

For the target population, a CVR greater than the estimated value of 0.35 was achieved for all items, obtaining a total CVR of 0.98 for the understanding criterion and 0.97 for clarity. However, the exclusion of item 4 was considered after reading the observations described by the target population about the content already included in item 3. For item 5, the sentence “related to therapeutic procedures” was added and, as for item 23, the inclusion of the term “service protocol” was recommended. The adjustments made followed the suggestions of making the items more specific, without changing the meaning of the statement.

When assessing item 10, there was a writing reordering, suggested by the target population, with the intention of obtaining greater content clarity and understanding. Thus, the IPE-APH version consisted of 44 items with evidence of validity in terms of content.

## DISCUSSION

In this study, evidence was sought for constructing IPE-APH items that expressed the EP phenomenon in MPHC through judges’ and the target population’s collaboration considering that content validity is a complex and rigorous stage, essential for developing health measurement instruments^(24)^.

Thus, the new instrument consists of a total of 44 items, distributed across four dimensions: Ethical problems arising from relationships with users and/or family members at the time of care provided by MPHC teams; Intra and inter-team ethical problems of RUE components; Ethical problems related to the organizational structure of RUE components; Ethical problems related to external factors involved in the MPHC scenario.

The conceptual matrix was a guiding element for the operational construction of dimensions and items, through literature review and rigorous assessment by the research team, as indicated in the literature^(12)^. It clearly highlights the differences between ethical conflicts with extremist solutions and described as dilemmas in relation to the repertoire of possibilities that can emerge when we guide and consider EP occurrence in clinical bioethics^(25)^.

The multidimensionality proposed for IPE-APH was based on instruments measuring the construct tested in primary care^(6-26)^. The specificities of dimensions 3 and 4 were added, placing the MPHC as a component that integrates the emergency care network and external factors that translate EPs occurring in the street scenario, according to theoretical studies carried out in Brazil and Sweden, respectively^(27-28)^.

Thus, these dimensions support that EPs are not only related to procedural issues, but occur in various relationships. It is necessary to consider in the world of relationships the essence of human beings and the ethical conflicts that involve values, norms and professional duties^(29)^.

In the content validity stage process, the qualitative and quantitative assessment of items becomes essential as a way of ensuring whether the list of responses to the latent variable is appropriate from a psychometric point of view^(9)^.

The instrument overall assessment presented a CVR greater than 0.45 for all dimensions, and in relation to the items, the predominance of a value higher than the estimated critical CVR^(21)^ for the judgment of judges and the target population. It is noted, then, that indexes were obtained that report sources of evidence of content validity for IPE-APH.

The hierarchical assessment of the relevance criterion on clarity allowed three items to remain, such as “Participation in procedures without user consent”, “Lack of active listening by the doctor to users’ complaints during care” and “Delay in the advanced support team’s dispatch to service due to the risk stratification process”, as they express conflicts of values and duties experienced by nurses in the MPHC scenario, and are essentially characterized as manifestos of the construct^(30)^.

The item “Pre-judgment by professionals when assisting users of alcohol and other drugs”, added after a suggestion from experts, portrays an everyday ethical phenomenon and is consistent with a theoretical study that addresses the need to highlight the stigma and discrimination that exist in clinical practice in healthcare services^(31)^.

In general, the instrument was considered by MPHC professionals to be relevant, with a clear and comprehensive approach to the field of professional practice. To this end, the restructured and assessed items were in accordance with suggestions/guidance from experts, target population and research team, as recommended in the literature^(12)^.

The items that underwent adjustments with refinement of terms and words aimed to maintain instrument clarity and semantic adequacy for the target population, with the intention of minimizing risks of bias in the interpretability of theoretical content and inferences in subsequent statistical analyzes^(32)^.

IPE-APH can be used in the future as a tool that measures ethically problematic situations emerging in the street scene in SAMU’s daily practice. Study reports that there is a clear need to create ways for nurses to have the opportunity in their practice to reflect on EPs with a multidisciplinary team and recognize how much this practice implies the quality of healthcare^(8)^.

However, a greater accumulation of evidence guarantees arguments regarding instrument validity, especially when construct measurement in the researched context is still limited^(32)^. Thus, IPE-APH is an advancement and innovation for measuring the phenomenon in the pre-hospital scenario dynamism.

### Study limitations

Aware that carrying out content validity is just one of the stages in the process of developing an instrument, the need for other sources of evidence is identified as a limitation of this study. It is therefore suggested that the research continue with the extension of techniques and procedures that can make the instrument replicable in the MPHC population and in other practice contexts, aiming to obtain other measurements of the studied phenomenon.

### Contributions to nursing and health

IPE-APH, after being tested in the practical field, may contribute to the early identification of EPs, considering that contact with this tool will encourage creating spaces for dialogue, meetings as well as simulations between nurses and teams that will favor the prudent decision-making at MPHC.

## CONCLUSIONS

Based on the theoretical matrix, scoping review and the contribution of SAMU nurses and judges, it was possible to construct IPE-APH, structured in four dimensions and 44 items, which presents evidence of content validity. This is the first Brazilian instrument that addresses this construct in the context of MPHC, following contemporary standards that underlie psychometric studies.

However, the scarcity of studies on instruments that measure the phenomenon reinforces the need for new research to build lightweight technologies that enable the qualification of assistance offered by nurses and the MPHC nursing team. It is also important to highlight the importance of expanding the theme with an approach to essential methodological stages for developing instruments to obtain valid psychometric properties and applicability in nursing.

## References

[1] Torabi M, Borhani F, Abbaszadeh A, Atashzadeh-Shoorideh F. (2018). Experiences of pre-hospital emergency medical personnel in ethical decision-making: a qualitative study. BMC Med Ethics.

[2] Zoboli E. (2012). Clinical bioethics in diversity: the essential contribution of the deliberative proposal of Diego Gracia. Rev Bioethik.

[3] Silveira LR, Ramos FR, Schneider DG, Razquin MIS, Brehmer LC. (2019). Processo de deliberação moral dos enfermeiros de competência gerencial e fiscalizatória dos conselhos de enfermagem. Enferm Foco.

[4] Bremer A, Holmberg M. (2020). Ethical conflicts in patient relationships: experiences of ambulance nursing students. Nurs Ethics.

[5] Silva LT. (2008). Construção e validação de um instrumento para avaliação de ocorrência de problema ético na Atenção Básica.

[6] Santos DV, Freitas KS, Rosa DOS, Zoboli ELCP, Miranda JOF (2021). Dimensional validity of the inventory of ethical problems in primary health care in the context of children’s health. Texto Contexto Enferm.

[7] Junges JR, Zóboli ELCP, Schaefer R, Nora CRD, Basso M. (2014). Validation of the comprehensiveness of an instrument on ethical problems in primary care. Rev Gaúcha Enferm.

[8] Nora CRD, Zoboli E, Vieira MM. (2017). Moral sensitivity of nurses assessed through scoping review. Cogitare Enferm.

[9] American Educational Research Association, American Psychological Association, National Council on Measurement in Education (2014). Standards for Educational and Psychological Testing.

[10] Oliveira SS. (2022). Construção e validação de um inventário de problemas éticos vivenciados por enfermeiras no atendimento pré-hospitalar móvel.

[11] Khodyakov D, Hempel S, Rubenstein L, Shekelle P, Foy R, Salem-Schatz S (2011). Conducting online expert panels: a feasibility and experimental replicability study. BMC Med Res Methodol.

[12] Devellis RF. (2017). Scale Development: theory and applications.

[13] Gong Q, Yang H. (2018). Balance of opinions in expert panels. Econ Lett.

[14] Gracia D. (2011). La alianza deliberativa. Bioét Complut.

[15] Oliveira SS, Pitzer CMT, Ferraz MOA, Lírio LKS, Santa Rosa DOS, Freitas KS (2022). Ethical problems in the clinical practice of mobile pre-hospital care nurses: a scoping review. Online Braz J Nurs.

[16] Hungerbünler I, Wang YP., Gorestein C, Wang YP, Hungerbühler I (2016). Instrumentos de avaliação em saúde mental.

[17] Souza VR, Marziale MH, Silva GT, Nascimento PL. (2021). Translation and validation into Brazilian Portuguese and assessment of the COREQ checklist. Acta Paul Enferm.

[18] Reichenheim M, Bastos JL. (2021). What, what for and how? developing measurement instruments in epidemiology. Rev Saude Publica.

[19] Malta M, Cardoso LO, Bastos FM, Magnanini MMF, Silva CMFP. (2010). STROBE iniciative: guidelines on reporting observational studies. Rev Saude Publica.

[20] Peters MDJ, Godfrey C, McInerney P, Munn Z, Tricco AC, Khalil H., Aromataris E, Munn Z (2020). JBI Manual for Evidence Synthesis.

[21] Baghestani AR, Ahmadi F, Tanha A, Meshkat M. (2017). Bayesian Critical Values for Lawshe’s Content Validity Ratio. Meas Eval Couns Dev.

[22] Almanasreh E, Moles R, Chen TF. (2019). Evaluation of methods used for estimating content validity. Res Social Adm Pharm.

[23] Nobile GG, Barrera SD, Rebustini F. (2021). Avaliação da alfabetização: elaboração e validação de conteúdo do IBALEC. Rev Psicopedag.

[24] Sireci S, Faulkner-Bond M. (2014). Validity evidence based on test content. Psicothema.

[25] Zoboli E. (2016). A aplicação da deliberação moral na pesquisa empírica em bioética. Rev Iberoam Bioét.

[26] Junges JR, Zoboli ELCP, Patussi MP, Schaefer R, Nora CRD. (2014). Construção e validação do instrumento “Inventário de problemas éticos na atenção primária em saúde”. Rev Bioét.

[27] Oliveira SS, Santos DV., Silva MSRM. (2021). Yellowbook Enfermagem: fluxos e condutas em urgência e emergência.

[28] Bijani M, Abedi S, Karimi S, Tehranineshat B. (2021). Major challenges and barriers in clinical decision-making as perceived by emergency medical services personnel: a qualitative content analysis. BMC Emerg Med.

[29] Mattozinho FCB, Freitas GF. (2021). Analysis of ethical issues: criminal acts in nursing practice. Acta Paul Enferm.

[30] Oliveira SS, Lima AB, Santa-Rosa DO, Freitas GF, Ferraz MOA. (2020). Experiences of the moral deliberation of nurses in mobile pre-hospital care. Rev Baiana Enferm.

[31] Godoi AMM, Garrafa V. (2014). Bioethics reading of the principle of non-discrimination and non-stigmatization. Saúde Soc.

[32] Bandalos DL. (2018). Measurement Theory and Applications for the Social Sciences.

