# Supplementary material for: Inventory of ethical problems in mobile pre-hospital care
Source: Rev Bras Enferm. 2025 Jan 10;77(Suppl 4):e20230539. doi: 10.1590/0034-7167-2023-0539 (PMC11726900; doi:10.1590/0034-7167-2023-0539)
Supplement: Translation

# Translation: Inventário de problemas éticos no atendimento pré-hospitalar móvel

**DOI:** 10.1590/0034-7167-2023-0539pt

**Authors:** Simone da Silva Oliveira, Mariana Oliveira Antunes Ferraz, Darci de Oliveira Santa Rosa, Kátia Santana Freitas, Elaine de Oliveira Souza, Deisy Vital dos Santos, Marisa Aparecida Amaro Malvestio

**Affiliations:** I Universidade Federal da Bahia. Salvador, Bahia, Brasil; II Universidade Estadual do Sudoeste da Bahia. Jequié, Bahia, Brasil; III Universidade Estadual de Feira de Santana. Feira de Santana, Bahia, Brasil; IV Universidade Federal do Recôncavo da Bahia. Santo Antônio de Jesus, Bahia, Brasil; V Universidade de São Paulo. São Paulo, São Paulo, Brasil

**Keywords:** Estudos de Validação, Bioética, Ética, Enfermeiras e Enfermeiros, Socorro de Urgência

## Abstract

**Objetivo:**

construir e validar conteúdo de um inventário de problemas éticos vivenciados por enfermeiras no atendimento pré-hospitalar móvel.

**Método:**

estudo com abordagem psicométrica, desenvolvido com as seguintes etapas: (1) construção do instrumento através de uma matriz teórica fundamentada na bioética deliberativa, revisão de escopo e pesquisa qualitativa *online*; (2) validade de conteúdo por juízes; (3) pré-teste com enfermeiras do Serviço de Atendimento Móvel de Urgência de variados estados brasileiros. Para a análise de validade de conteúdo, calculou-se o *Content Validity Ratio* (CVR>0,45 para os juízes e CVR>0,35 para a população-alvo).

**Resultados:**

o instrumento contou com 44 itens, distribuídos em quatro dimensões.

**Considerações finais:**

o instrumento construído apresentou fontes de evidências de validade de conteúdo, conferindo boas medidas psicométricas e constituindo uma ferramenta útil para a prática de enfermeiras no cenário pré-hospitalar.

## INTRODUÇÃO

O atendimento pré-hospitalar móvel (APHM) é definido como a assistência que ocorre fora do ambiente intra-hospitalar por profissionais que compõem equipes de unidades móveis, em um processo de trabalho na perspectiva multidisciplinar, visando assegurar o atendimento de urgência e emergência às pessoas, de acordo com o grau de complexidade de cada caso. Nesse serviço, profissionais vivenciam desafios de cuidados dos usuários nas circunstâncias mais inusitadas e, por vezes, enfrentam prioridades difíceis entre valores conflitantes e normas^(1)^.

Devido ao contexto que incorre em imprevisibilidade das situações do atendimento, o cenário do APHM é convidativo para vivências inusitadas, cuja complexidade advoga para a necessidade da discussão sobre os problemas éticos (PEs) que emergem no cotidiano do trabalho das enfermeiras.

No âmbito da prática clínica, os conflitos éticos, geradores de indecisões entre os profissionais envolvidos no caso, podem ser percebidos como problemas ou dilemas. A diferenciação entre os PEs e os dilemas é que, no primeiro, encontram-se várias possibilidades para resolução do caso, enquanto que, no segundo, as decisões são sempre reduzidas a duas saídas extremas^(2)^. Ciente da pluralidade moral que envolve o agir profissional, as questões éticas pautadas como problemas traduzem o fenômeno com seus múltiplos sentidos éticos e a necessidade de diálogos e reflexões para a busca de soluções responsáveis na prática da enfermagem^(3)^.

Assim, PEs que envolvem o interesse do paciente, o ideário profissional, a estrutura organizacional/gerencial, as decisões durante a reanimação do usuário e a interferência de outras pessoas e/ou profissionais no momento do atendimento são descritos como frequentes no APHM^(4)^.

No Brasil, existem instrumentos que têm sido utilizados e adaptados para a medida de PEs na Atenção Primária. O Inventário de Problemas Éticos na Atenção Primária à Saúde (IPE-APS) foi estruturado pioneiramente em três dimensões que reportam o fenômeno vivenciado por profissionais médicos e enfermeiros^(5)^. Depois, foi adaptado para a medida do construto na área da saúde da criança e apresentou propriedades psicométricas válidas e confiáveis^(6)^.

Destaca-se que os instrumentos validados devem se apresentar não como uma mera descrição dos PEs, mas devem direcionar ações de transformação da prática através de evidências^(7)^. No entanto, apesar da relevância do fenômeno, não foram identificados na literatura estudos que abordem parâmetros de medida para identificar PEs no contexto do APHM, evidenciando uma lacuna do conhecimento.

Destarte, considera-se importante que sejam analisados aspectos culturais, morais e éticos intrínsecos ao fenômeno, levando-se em conta os níveis de atenção à saúde. Para tanto, ao se elaborar um instrumento de medida de PEs para o APHM, possibilita-se uma nova estrutura de equacionamento ético e processos decisórios na enfermagem.

Com isso, elaborar um instrumento apropriado para mensuração do problema ético em cenário de APHM pode favorecer a tomada de decisões éticas sensíveis e prudentes, bem como o desenvolvimento de competências ético-morais das enfermeiras que atuam nesse serviço de saúde^(8)^, potencializando a qualidade do atendimento em situações de emergência.

## OBJETIVO

Construir e validar conteúdo de um inventário de problemas éticos vivenciados por enfermeiras no atendimento pré-hospitalar móvel.

## MÉTODOS

### Aspectos éticos

Este estudo foi aprovado pelo Comitê de Ética em Pesquisa da Escola de Enfermagem da Universidade Federal da Bahia, e atende aos requisitos previstos na Resolução n^o^ 466/12 do Conselho Nacional de Saúde, que regulamenta pesquisa envolvendo seres humanos, e nota técnica, que assegura orientações para o desenvolvimento de pesquisa em ambiente virtual. Todas(os) as(os) participantes preencheram o Termo de Consentimento Livre e Esclarecido (TCLE).

### Desenho, local do estudo e período

Trata-se de estudo com abordagem psicométrica, fundamentado em recomendações da *American Educational Research Association* (AERA), *American Psychological Association* (APA), e do *National Council on Measurement in Education* (NCME)^(9)^. Desenvolveu-se, no período de outubro de 2021 a abril de 2022, em três etapas: (1) elaboração do instrumento através da matriz teórica, revisão de escopo e pesquisa qualitativa *online*; (2) validação do conteúdo por um painel de juízes; e (3) pré-teste com a população-alvo.

O estudo faz parte de uma tese de doutorado intitulada “Construção e validação de um inventário de problemas éticos vivenciados por enfermeiras no atendimento pré-hospitalar móvel”^(10)^, apresentada ao Programa de Pós-Graduação em Enfermagem e Saúde da Universidade Federal da Bahia. Diante do contexto da pandemia de COVID-19, a pesquisa se manteve no formato *online n*as etapas que envolviam participantes; para tanto, foram utilizadas ferramentas como *Google Forms* ^®^ *, WhatsApp* ^®^ e *e-mail*. Assegura-se, ainda, que essa modalidade tem sido proposta como propulsora de alcance de populações, principalmente para a condução de painéis de especialistas, quando esses estão geograficamente distribuídos em regiões distintas^(11)^.

### População e amostra: critérios de inclusão e exclusão

Na primeira etapa, realizou-se pesquisa qualitativa *online* por meio de uma amostra intencional, não probabilística, com representação de enfermeiras de cinco regiões do Brasil. Para acessar essa população, foi utilizado o grupo nacional de *WhatsApp* ^®^ de instrutores do *Pre-Hospital Trauma Life Support* (PHTLS) e instrutores do *Advanced Trauma Care for Nurses* (ATCN), do qual uma das autoras é integrante. As participantes deveriam ser enfermeira do Serviço de Atendimento Móvel de Urgência (SAMU), com um tempo mínimo de um ano de atuação na área.

Para a segunda etapa, compôs-se um comitê de juízes, atendendo aos critérios de ser enfermeira com experiência na área de urgência e emergência pré-hospitalar, com foco na gestão, pesquisa, ensino, assistência direta, ou que tivesse aproximação com o construto PEs e/ou possuísse a *expertise* na validação de instrumentos na prática da saúde. A seleção dessa população ocorreu por meio de busca simples na Plataforma *Lattes*, utilizando as palavras-chave “Bioética”, “Atendimento Pré-Hospitalar Móvel” e “Nacionalidade Brasileira”. Também foi utilizado o grupo nacional de *WhatsApp* ^®^ de instrutores do PHTLS e instrutores do ATCN. Para maior alcance do número de participantes em uma possibilidade de falta de consenso no julgamento do instrumento, considerou-se uma composição heterogênea para essa etapa^(12)^. Um número acima de dez juízes, como associável a uma confiabilidade aceitável^(13)^, atendeu à complexidade para o desenvolvimento de itens e dimensões.

Por fim, na etapa 3, foi selecionada, por conveniência, a população-alvo de enfermeiras, integrantes das equipes de intervenção do SAMU do país.

### Percurso teórico-metodológico

A primeira etapa do estudo foi constituída por fundamentação teórica e conceitual do construto, apoiado no referencial teórico-metodológico da deliberação moral de Diego Gracia, para a construção da matriz teórica, por considerar os conflitos éticos como problemas e fundamentar que a realidade apresenta uma pluralidade moral complexa com proposta de alternativas prudentes capazes de valorar o que está envolvido em uma situação clínica^(14)^.

Para a etapa de construção das dimensões e itens, foi realizada revisão de literatura no formato de *scoping review* com o objetivo de mapear a evidência científica sobre os PEs vivenciados por enfermeiras no APHM. Foi utilizado o modelo proposto pelo JBI para a condução de todas as fases dessa etapa. Para tanto, registrou-se a revisão no *Open Science Framework* (OSF) com registro DOI: 10.17605/OSF.IO/PS9RA^(15)^. Contudo, os metadados foram acessados no período de outubro a novembro de 2021 nas bases de dados MEDLINE/PubMed, Literatura Latino-Americana e do Caribe em Ciências da Saúde (LILACS), SAGE *Journals, Cumulative Index to Nursing and Allied Health* (CINAHL) e *Web of Science*. Foram considerados os Descritores em Ciências da Saúde (DeSC), o *Medical Subject Headings* (MeSH) e as palavras-chave *“nursing”, “nurse”, “prehospital”, “ethics”, “emergency relief”, “bioethics”, “ethical dilemmas”, “prehospital”, “ambulances”*, combinados entre si com os operadores booleanos “*AND*” e “*OR*”. Foram incluídos os textos científicos disponibilizados na íntegra, sem restrição de desenho e de idiomas^(15)^.

Na etapa 1, na pesquisa qualitativa, realizada nos meses de novembro e dezembro de 2021, houve aplicação de um questionário *online* considerando o controle de *etic* (observações do pesquisador sobre o construto) e *emic* (observações das pessoas locais sobre o construto), para a formulação dos itens em estudos de validação através da participação da população que vivencia o fenômeno^(16)^. A coleta foi realizada por um questionário, por meio do aplicativo *Google Forms^®^,* composto por instruções para o preenchimento do questionário, dados referentes à caracterização pessoal e profissional e à apresentação das dimensões, além de itens evidenciados na revisão de literatura, para posterior identificação de sua ocorrência pelos respondentes através de múltipla escolha. Optou-se, ainda, por um espaço de texto para os participantes descreverem outros PEs vivenciados na prática do APHM. O relatório desta etapa foi norteado pelo *COnsolidated criteria for REporting Qualitative research* (COREQ)^(17)^.

De posse dessas informações, um grupo de pesquisa composto por três pesquisadoras com *expertise* nas áreas da ética, bioética e desenvolvimento de instrumentos em saúde analisou cada item e dimensão para a estruturação do instrumento.

Para a etapa 2, foi contemplado o processo de validação do conteúdo. Buscou-se avaliar a abrangência das dimensões, bem como a clareza, a relevância e a avaliação geral dos itens, obtendo-se opinião de juízes sobre a adequação semântica e operacional do instrumento. Foram também utilizados um formulário eletrônico via *Google Forms^®^* com instruções gerais para os participantes, formulário sobre caracterização pessoal, profissional, acadêmica das respondentes, bem como questões inicias relacionadas à avaliação das dimensões. Considerou-se o critério para a abrangência, ao se avaliar se todos os itens contemplavam as dimensões pré-definidas e representavam a variável latente.

Quanto aos itens, a avaliação ocorreu individualmente, através do julgamento dos juízes quanto ao critério de relevância (observar se os itens refletiam um comportamento que expressasse o problema ético que se pretende medir) e clareza (verificar se a redação do item quanto à sua compreensibilidade). As respostas seguiram o tipo de escala dicotômica; para essa etapa do estudo, utilizou-se pontuação zero para respostas “não” e pontuação um para respostas “sim”. Possibilitou-se, ainda, a avaliação geral do instrumento pautando os elementos: apresentação dos itens e dimensões; orientações para a população-alvo; e a escala de resposta proposta para o instrumento. Atendeu-se ao requisito de ser essencial à avaliação do instrumento o cenário operacional em sua fase de desenvolvimento^(18)^.

Após os ajustes do instrumento, realizou-se a condução da etapa 3, através do pré-teste com a população-alvo, que ocorreu no mês de abril 2022. Uma versão preliminar, com o conteúdo validado, foi testada por enfermeiras que avaliaram a clareza e a compreensão dos itens, além dos aspectos operacionais para a aplicabilidade do instrumento na população-alvo. O relatório das etapas quantitativas foi conduzido a partir dos itens do *STrengthening the Reporting of OBservational studies in Epidemiology* (STROBE)^(19)^.

### Análise dos resultados e estatística

Para a apresentação dos dados da *scoping review*, foi utilizado o PRISMA-Scr, que oferece clareza e transparência na apresentação dos dados da revisão de escopo realizada^(20)^.

Para organização e análise, os dados das etapas do estudo quantitativo foram tabulados no programa *Microsoft Excel* ^®^, e depois avaliados. No processo de análise da validação de conteúdo, calculou-se o *Content Validity Ratio* (CVR), criado por Lawshe e calculado através da fórmula CVR = (n_e_ - N/2) / (N/2), sendo que n_e_ é o número de avaliadores que julgou de forma positiva (claro/relevante) o item e N é a quantidade de juízes participantes do estudo^(21)^. O índice tem sido indicado pela literatura por não sofrer inflação dos dados com a adequação do número de especialistas que compõem o painel^(22)^.

Considerou-se, assim, como ponto de corte o CVR 0,45, sendo os itens com valores menores avaliados e modificados ou excluídos, conforme recomendações dos juízes.

O programa *Statistical Package for the Social Sciences* (SPSS), versão 22.0, foi utilizado para análise das variáveis contínuas quanto às medidas de tendência central (média) e dispersão (desvio padrão), e a caracterização dos participantes foi apresentada pela frequência relativa.

## RESULTADOS

Os resultados da primeira etapa do estudo correspondem à construção da matriz teórica do construto PE, obtido através da revisão da literatura e fundamentação teórica da bioética deliberativa^(14)^. Assim, ficou proposto um marco conceitual (Figura 1), que foi revisitado pelas pesquisadoras durante todas as etapas de desenvolvimento, refinamento e ajustes de dimensões e itens.

**Figura 1:** Estrutura conceitual de problemas éticos na prática clínica da enfermeira

Fez-se a leitura das categorias, obtidas com a revisão e o aporte teórico, considerando os conflitos de valores e deveres balizados no marco conceitual da deliberação moral para a descrição dos PEs encontrados no cenário do APHM e conseguinte construção das dimensões e itens.

A *scoping review* alcançou nove textos, fundamentando a primeira etapa do desenvolvimento do instrumento. Assim, quatro dimensões emergiram, a saber: Dimensão 1: Problemas éticos oriundos das relações com usuários e/ou familiares no momento do atendimento realizado pelas equipes do APHM; Dimensão 2: Problemas éticos intra e interequipes dos componentes das Rede de Atenção as Urgências e Emergências (RUE); Dimensão 3: Problemas éticos relacionados a estrutura organizacional dos componentes da RUE; Dimensão 4: Problemas éticos relacionados aos fatores externos intervenientes no cenário do APHM^(15)^.

Em consonância, foram ampliados ao quadro operacional 12 itens, descritos pela população-alvo durante a autoaplicação do questionário da pesquisa *online*. Definiu-se, assim, uma estrutura com 55 itens para o instrumento construído denominado Inventário de Problemas Éticos vivenciados por enfermeiras no Atendimento Pré-Hospitalar Móvel (IPE-APH).

Destarte, a primeira versão do inventário passou pela etapa de validação de conteúdo por 22 especialistas, sendo esses na maioria do gênero feminino (72,7%), com titulação acadêmica distribuída entre mestres (31,8%), doutores (31,8%) e especialistas (31,8%). Apresentaram as médias de idade de 43,7 anos (DP = 8,4), tempo de formação de 18,8 anos (DP = 8,2) e tempo de atuação no APHM de 17,4 anos (DP = 7,6). Os locais de atuação profissional identificados foram dez estados brasileiros, como Acre, Bahia, Goiás, Pará, Paraná, Pernambuco, Rio Grande do Sul, Santa Catarina, São Paulo, Sergipe, e o Distrito Federal. Desses, possuem experiência na assistência (72,7%), docência (72,7%), gerência (31,8%) e pesquisa (54,5%) nas áreas de atendimento pré-hospitalar, ética, bioética ou instrumento de medida.

Durante a análise dos dados obtidos com a avaliação, as quatro dimensões tiveram um valor de CVR crítico de 0,73 para a abrangência, caracterizando a cobertura do conjunto de PEs pela variável latente. Realizou-se, ainda, o cálculo do CVR para os itens, conforme a Tabela 1.

**Tabela 1:** *Content Validity Ratio* para seleção dos itens do Inventário de Problemas Éticos vivenciados por enfermeiras no Atendimento Pré-Hospitalar Móvel quanto à relevância e clareza, Brasil, 2022 (n=22)

Dimensão 1: Problemas éticos oriundos das relações com usuários e/ou familiares no momento do atendimento realizado pelas equipes do APHM			
**Itens**		CVR^*^	
		**R**	**C**
1	Recusa do usuário em ser atendido por profissionais do sexo oposto ao seu.	0,55	0,91
2	Recusa do usuário em ser transportado pela equipe do APHM.	0,55	0,82
3	Participação em procedimentos sem o consentimento do usuário.	0,91	0,27
4	Desrespeito ao direito de escolha do usuário.	0,91	0,73
5	Desrespeito ao direito de escolha da família.	0,91	0,64
6	Omissão de informações sobre a ocorrência, por parte de familiares.	0,73	0,64
7	Dificuldade em proteger informações sigilosas, no atendimento aos usuários.	0,91	0,73
8	Dificuldade em manter a privacidade do usuário durante o atendimento.	0,91	0,73
9	Descaso com a manutenção do suporte à vida em caso de usuário com mau prognóstico.	1,0	0,64
10	Ausência de informações para o usuário que justifique ações do atendimento profissional.	0,82	0,55
11	Falta de escuta ativa do Enfermeiro, às queixas do usuário durante o atendimento	0,73	0,73
12	Falta de escuta ativa do Médico, às queixas do usuário durante o atendimento.	0,91	0,36
13	Julgamento de Enfermeiros do APHM sobre aspectos religiosos que constrange o usuário.	1,0	0,55
14	Julgamento de Enfermeiros do APHM sobre aspectos sexuais que constrange o usuário.	0,91	0,55
15	Julgamento de Enfermeiras do APHM sobre aspectos de gênero que constrange o usuário.	0,82	0,73
16	Julgamento de Enfermeiros do APHM sobre aspectos socioeconômicos que constrange o usuário.	0,82	0,45
17	Preconceito étnico racial por parte de Enfermeiros do APHM.	1,0	0,55
18	Preconceito a vítimas de tentativa de suicídio por parte de Enfermeiros do APHM.	1,0	0,91
19	Desrespeito entre as equipes do atendimento pré-hospitalar móvel e das unidades de referência.	1,0	0,91
**Dimensão 2:** Problemas éticos intra e interequipes dos componentes das Rede de Atenção às Urgências e Emergências (RUE)			
**Itens**		CVR^*^	
		**R**	**C**
20	Desrespeito entre o Enfermeiro e demais componentes do APHM	1,0	0,91
21	Dificuldade de comunicação da Enfermeira com as demais profissionais da equipe do APHM.	0,73	0,73
22	Dificuldade de comunicação entre os membros que compõem as equipes do atendimento pré-hospitalar móvel e das unidades de referência.	0,91	0,82
23	Descumprimento de protocolo por parte das equipes do APHM.	0,91	0,64
24	Desacordo entre médicos reguladores e membros da equipe de intervenção do APHM.	0,64	0,64
25	Falta de empatia por parte dos profissionais que compõem a central de regulação das urgências.	0,36	0,55
26	Questionamento dos membros da equipe de suporte avançado, quanto à conduta médica oriunda da regulação.	0,55	0,55
27	Despreparo técnico por parte dos profissionais que compõem as equipes do APHM.	0,64	0,64
28	Despreparo dos profissionais médicos que compõem o Intra-hospitalar durante a transferência de cuidados	0,45	0,55
29	Dificuldade do Enfermeiro em priorizar o atendimento a múltiplas vítimas em situação de desastres.	0,55	0,27
30	Discordância das condutas médicas quando compromete a segurança do usuário.	0,82	0,55
31	Omissão de conduta da equipe médica durante o atendimento.	0,73	0,64
32	Falta de compromisso dos profissionais com o serviço do APHM.	0,73	0,55
33	Dificuldade de colaboração entre os profissionais que compõem a equipe de intervenção.	0,73	0,73
34	Discordância na condução terapêutica entre os médicos reguladores e os membros da equipe de intervenção do APHM.	0,64	0,64
35	Omissão no registro de procedimentos realizados com o usuário, por parte dos profissionais das equipes do APHM.	0,91	0,73
**Dimensão 3:** Problemas éticos relacionados a estrutura organizacional dos componentes da RUE			
**Itens**		CVR^*^	
		**R**	**C**
36	Remoções de pacientes sem indicação de atendimento emergencial.	0,55	0,64
37	Retardo no disparo da equipe de suporte avançado para o atendimento em função do processo de classificação de risco.	0,73	0,27
38	Indisponibilidade de leitos nas unidades de referência.	0,55	0,82
39	Recusa na admissão dos usuários por parte das equipes do Intra-hospitalar.	0,91	0,91
40	Dificuldade no sistema de telecomunicação entre a central de regulação das urgências e a equipe de intervenção.	0,45	0,82
41	Envio das unidades móveis para ocorrências sem gravidade.	0,55	0,73
42	Escassez de equipamentos para os atendimentos ao usuário.	0,55	0,73
43	Escassez de insumos para os atendimentos ao usuário.	0,73	0,73
44	Desfalque das equipes que compõem a Unidade de Suporte Avançado.	0,73	0,82
45	Retenção de equipamentos das unidades móveis junto aos usuários admitidos nas unidades de referência.	0,73	0,82
46	Retardo no tempo-resposta do atendimento ao usuário em função do desfalque da frota.	0,91	0,82
Dimensão 4: Problemas éticos relacionados aos fatores externos intervenientes no cenário do APHM			
**Itens**		CVR^*^	
		**R**	**C**
47	Desrespeito à preservação da imagem da equipe por meio de fotos obtidos durante o atendimento.	0,91	0,91
48	Desrespeito à preservação da imagem da equipe por meio de vídeos obtidos durante o atendimento.	0,64	0,64
49	Retardo no atendimento ao usuário em função da interferência de populares na cena da ocorrência.	0,73	0,73
50	Interferência no atendimento ao usuário em função da ação de outros profissionais da saúde presentes na cena.	0,73	0,82
51	Interferência no atendimento ao usuário em função de familiares presentes na cena.	0,73	0,73
52	Dificuldade no atendimento ao usuário devido ao retardo do apoio Policial na cena	0,55	0,82
53	Dificuldade no atendimento ao usuário devido ao retardo do apoio de corpo de bombeiros na cena.	0,36	0,73
54	Retardo no atendimento ao usuário devido ao tempo dispensado para o boletim de ocorrência policial.	0,36	0,64
55	Exposição a situações que ameaçam a vida da equipe no local da ocorrência.	0,55	0,73

* *Content Validity Ratio; R = relevância; C = clareza.*

Devido ao número de itens que obtiveram um CVR <0,45 na avaliação do critério de relevância e clareza, optou-se por uma avaliação hierárquica, priorizando a exclusão dos itens não relevantes, e a clareza foi definida em conjunto com uma análise aprofundada da teoria que subsidia o instrumento para manter, alterar ou excluir o item, conforme estudo psicométrico validado no Brasil^(23)^.

Em relação aos itens, observa-se que a maioria alcançou valor maior que 0,45, o que atende ao valor recomendado na literatura, conforme o número de juízes^(21)^. Sete itens não obtiveram o valor estimado, sendo, entre estes, quatro eliminados (25, 29, 53 e 54) e três avaliados (3, 13, 37).

Durante a avaliação extensiva do instrumento, os itens 13 e 37 foram considerados pela equipe de pesquisa como relevantes para o instrumento e, logo, mantidos. O item 1 teve a sentença “profissionais do sexo oposto ao seu” substituída por “profissionais do APHM”. Já o item 18 foi ajustado com a inclusão de “ou em sofrimento psíquico”, sugerida pelos juízes. Enquanto o item 3, mesmo com um CVR 0,91 para relevância e 0,27 para clareza, sofreu um ajuste de termos com a substituição da palavra “participação” por “realização”.

O item 47 sofreu modificação com a junção das palavras “vídeos ou fotos”, atendendo à sugestão dos juízes e leitura da equipe de pesquisa com a compreensão de que, independentemente do meio utilizado, obtém-se o desrespeito à preservação da imagem da equipe, como PE. Neste sentido, o item 48 foi contemplado e excluído do instrumento.

Os itens 21, 22, 26, 30 e 31, apesar dos índices satisfatórios, foram considerados já contemplados, sendo retirado da versão do instrumento. O item 28 foi ajustado com a substituição dos termos “despreparo dos profissionais médicos” por “despreparo técnico dos profissionais”. O item 35 teve a palavra “omissão” substituída pelo termo “registro insuficiente”.

Durante a análise, foi acrescentado o item “Pré-julgamento por parte dos profissionais no atendimento ao usuário de álcool e outras drogas”, contemplando as orientações dos juízes quanto à ocorrência frequente desse PE no cotidiano do APHM.

Mesmo com os ajustes e refinamento dos itens, o instrumento manteve-se com um CVR total de 0,81 para a relevância e 0,71 para a clareza.

Assim, após as alterações sugeridas pelos juízes, o IPE-APH foi estruturado com 45 itens e submetido à avaliação da população-alvo. Participaram, desta etapa, 32 enfermeiras (65,6%), em sua maioria com titulação acadêmica de especialista (68,8%). Apresentaram as médias de idade de 40,6 anos (DP = 6,6), tempo de formação de 14,2 anos (DP = 7,1) e tempo de atuação no APHM de 9,6 anos (DP = 4,7). As participantes se declararam em maioria negras (31,3% pardas e 21,9% pretas), e a Unidade de Suporte Avançado foi o tipo de unidade de lotação de maior atuação das profissionais (81,3%).

Para a população-alvo, foi alcançado um CVR maior que o valor estimado de 0,35 para todos os itens, obtendo um CVR total de 0,98 para o critério de compreensão e 0,97 para a clareza. No entanto, considerou-se a exclusão do item 4 após leitura das observações descritas pela população-alvo sobre o conteúdo já contemplado no item 3. Para o item 5, foi adicionada a sentença “relacionado aos procedimentos terapêuticos” e, quanto ao item 23, foi recomendada a inclusão do termo “protocolo de atendimento”. Os ajustes realizados seguiram as sugestões de tornar os itens mais específicos, sem alteração do significado do enunciado.

Na avaliação do item 10, houve um reordenamento da escrita, sugerida pela população-alvo, com a intenção de obter maior clareza e compreensão do conteúdo. Assim, a versão do IPE-APH foi composta por 44 itens com evidências de validade quanto ao conteúdo.

## DISCUSSÃO

Buscaram-se neste estudo evidências para a construção de itens do IPE-APH que expressassem o fenômeno PE no APHM através da colaboração de juízes e da população-alvo, considerando que a validação de conteúdo se situa como uma etapa complexa e rigorosa, essencial para o desenvolvimento de instrumentos de medida em saúde^(24)^.

Assim, o novo instrumento é composto por um total de 44 itens, distribuídos em quatro dimensões: Problemas éticos oriundos das relações com usuários e/ou familiares no momento do atendimento realizado pelas equipes do APHM; Problemas éticos intra e interequipes dos componentes da RUE; Problemas éticos relacionados a estrutura organizacional dos componentes da RUE; Problemas éticos relacionados aos fatores externos intervenientes no cenário do APHM.

A matriz conceitual foi um elemento condutor para a construção operacional de dimensões e itens, através da revisão de literatura e avaliação rigorosa da equipe de pesquisa, conforme indicações da literatura^(12)^. Destaca claramente as diferenças entre os conflitos éticos com soluções extremistas e descritos como dilemas em relação ao repertório de possibilidades que podem emergir quando pautamos e consideramos a ocorrência de PE na bioética clínica^(25)^.

A multidimensionalidade proposta para o IPE-APH foi fundamentada em instrumentos de medida do construto testados na atenção primária^(6-26)^. As especificidades das dimensões 3 e 4 foram acrescidas, situando o APHM como um componente que integra a rede de atenção às urgências e os fatores externos que traduzem PEs ocorrentes no cenário de rua, conforme estudos teóricos realizados no Brasil e na Suécia, respectivamente^(27-28)^.

Assim, essas dimensões sustentam que os PEs não estão relacionados apenas às questões procedimentais, mas ocorrem nas diversas relações. Faz-se necessário considerar no mundo das relações a essência do ser humano e os conflitos éticos que envolvem valores, normas e deveres profissionais^(29)^.

No processo de etapa de validação do conteúdo, torna-se fundamental a avaliação qualitativa e quantitativa dos itens como forma de garantir se o elenco de respostas à variável latente é apropriado do ponto de vista psicométrico^(9)^.

A avaliação geral do instrumento apresentou um CVR maior que 0,45 para todas as dimensões, e em relação aos itens, foi destacada também a predominância de um valor superior ao CVR crítico estimado^(21)^ para o julgamento dos juízes e da população-alvo. Nota-se, então, a obtenção de índices que reportam fontes de evidência de validade de conteúdo para o IPE-APH.

A avaliação hierárquica do critério de relevância sobre a clareza permitiu a permanência de três itens, como “Participação em procedimentos sem o consentimento do usuário”, “Falta de escuta ativa do médico às queixas do usuário durante o atendimento” e “Retardo no disparo da equipe de suporte avançado para o atendimento em função do processo de classificação de risco”, visto que expressam conflitos de valores e deveres vivenciados por enfermeiras no cenário do APHM, e se caracterizam essencialmente como manifestos do construto^(30)^.

O item “Pré-julgamento por parte dos profissionais no atendimento ao usuário de álcool e outras drogas”, acrescentado após sugestão dos especialistas, retrata um fenômeno ético cotidiano e coaduna com estudo teórico que aborda a necessidade de destacar o estigma e a discriminação existentes na prática clínica nos serviços de saúde^(31)^.

Em geral, o instrumento foi considerado pelos profissionais do APHM como relevantes, de abordagem clara e compreensiva para o campo da prática profissional. Para tanto, os itens reestruturados e avaliados estiveram em concordância com as sugestões/orientações dos especialistas, população-alvo e equipe de pesquisa, conforme proposta recomendada na literatura^(12)^.

Os itens que sofreram ajustes com o refinamento de termos e palavras objetivaram manter a clareza e adequação semântica do instrumento para a população-alvo, com a intenção de minimizar os riscos de viés de interpretabilidade do conteúdo teórico e inferências nas análises estatísticas posteriores^(32)^.

O IPE-APH pode ser utilizado futuramente como uma ferramenta que mensura no cotidiano prático do SAMU situações eticamente problemáticas emergentes no cenário de rua. Estudo reporta ser notória a necessidade de criar vias para que o enfermeiro tenha na sua prática a oportunidade de refletir sobre os PEs com uma equipe multidisciplinar e reconhecer o quanto essa prática implica a qualidade da assistência à saúde^(8)^.

No entanto, um maior acúmulo de evidências garante argumentos de validade do instrumento, principalmente quando a medida do construto no contexto pesquisado ainda é limitada^(32)^. Assim, o IPE-APH apresenta-se como um avanço e inovação para a medição do fenômeno no dinamismo do cenário pré-hospitalar.

### Limitações do estudo

Cientes que a realização da validação do conteúdo é apenas uma das etapas no processo de desenvolvimento de um instrumento, identifica-se como limitação desse estudo a necessidade de outras fontes de evidência. Sugere-se, assim, a continuidade da pesquisa com a extensão de técnicas e procedimentos que possam tornar o instrumento replicável na população do APHM e em outros contextos de prática, visando à obtenção de outras medidas do fenômeno estudado.

### Contribuições para as áreas da enfermagem e saúde

O IPE-APH, após ser testado no campo prático, poderá contribuir com a identificação precoce de PEs, considerando que o contato com essa ferramenta estimulará a constituição de espaços de diálogos, de reuniões, bem como de simulações entre enfermeiras e equipes que favorecerão a tomada de decisão prudente no APHM.

## CONCLUSÕES

A partir da matriz teórica, *scoping review* e da contribuição de enfermeiros do SAMU e juízes, foi possível construir o IPE-APH, estruturado em quatro dimensões e 44 itens, que apresenta evidências de validade de conteúdo. Trata-se do primeiro instrumento brasileiro que aborda esse construto no contexto do APHM, seguindo normas contemporâneas que fundamentam os estudos psicométricos.

Todavia, a escassez de estudos sobre instrumentos que mensure o fenômeno reforça a necessidade de novas pesquisas para construção de tecnologias leves que possibilitem a qualificação da assistência ofertada pelas enfermeiras e pela equipe de enfermagem do APHM. Ressalta-se, ainda, a importância de ampliação da temática com abordagem de etapas metodológicas essenciais para a elaboração de instrumentos com obtenção de propriedades psicométricas válidas e aplicabilidade na área da enfermagem.
